# The complete mitochondrial genome of *Conger erebennus* (Jordan & Snyder, 1901) (Anguilliformes: Congridae) from Liaoning Province, China

**DOI:** 10.1080/23802359.2022.2135394

**Published:** 2022-10-27

**Authors:** Long Yan, Weikuang Wang, Jie Liu, Dong Guo

**Affiliations:** a Liaoning Ocean and Fisheries Science Research Institute, Dalian, China; b Modern Agricultural Production Development Service Center of Dalian, Dalian, China; c Department of Environmental Engineering and Science, Feng Chia University, Taichung, Taiwan; d Shandong Marine Forecast and Hazard Mitigation, Qingdao, China

**Keywords:** Mitogenome, *Conger erebennus*, phylogenetic

## Abstract

This study aims to explore the complete mitochondrial genome and phylogenetic relationship of *Conger erebennus* (Jordan & Snyder, 1901) (Anguilliformes: Congridae). The information about the mitogenome was obtained using Illumina’s next-generation sequencing technologies. The results revealed that the length of the circular mitogenome was 18,777 bp and it contained 37 genes and three unidentical control regions (CRs). The mitogenome contained 62.78% AT bases with an anti-G bias similar to that in most marine fishes. The maximum-likelihood phylogenetic tree of Anguiliformes based on encoding genes revealed that *C. erebennus* was in the same clade as other species of Congridae. The results of this study will progress the future of evolutionary research and species identification.

*Conger erebennus* (Jordan & Snyder, 1901) (Anguilliformes: Congridae) is mainly found in the Japanese waters of the south of Hokkaido and the Korean Peninsula (Odani et al. 2005), and was first reported from the coastal waters of China in the present study. *C. erebennus* is a rare nocturnal rare deep-sea fish occasionally caught by fishing nets. During the day, it hides in caves within sandy-mud substrates. Partial molecular sequence analyses of 16S rRNA (GenBank accession number: AB922176.1) and 12S rRNA (GenBank accession number: LC421664.1) have been performed on *C. erebennus*; however, the complete mitogenome remains undeciphered. In this study, we have revealed the circular mitogenome and phylogenic relationship of a *C. erebennus* specimen.

The specimen of *C. erebennus* (no. LHYT2020-021) was collected from Zhangzi Island in Changhai County, north of the Yellow Sea (122°01′E, 39°10′N), on 20 September 2021, and was kept in the museum of Liaoning Ocean and Fisheries Science Research Institute (Dong Guo, guohongtiansky@163.com). The total genomic DNA of the muscle tissue was extracted by the modified cetyltrimethylammonium bromide (CTAB) method (Sambrook and Russell 2001). NEBNext Ultra DNA Library Prep Kit was used to construct the 500-bp paired-end DNA library for Illumina sequencing. The complete mitogenome was deciphered by Illumina NovaSeq 6000 sequencing platform. The mitogenome of *C. erebennus* was assembled and annotated using the online MITOS tool, with the mitogenome of a closely related species, *Conger japonicus* (GenBank accession number: NC_027186.1), as reference.

The circular mitogenome of *C. erebennus* is 18,777 bp long (GenBank accession number: OM691699), making it the longest mitogenome sequence published from the order Anguilliformes (Zhang et al. 2021). The gene component of its mitogenome is highly similar to that of most vertebrates (Boore 1999). It contains 12S rRNA, 16S rRNA, 13 protein-coding genes, 22 tRNA genes, and three control regions (CRs). *ND6* and eight tRNA genes (*Ala*, *Asn*, *Cys*, *Gln*, *Glu*, *Ser-UCN*, *Tyr*, and *Pro*) are encoded on the L-strand, while other mitochondrial genes are encoded on the H-strand. The mitochondrial nucleotide composition of *C. erebennus* is 33.62% A, 29.16% T, 22.11% C, and 15.11% G. The mitochondrial base composition of *C. erebennus* has an anti-G bias like most marine fishes (Miya et al. 2003), and a slight excess of AT (62.78%). The initiation codon for 11 protein-coding genes (*ATP8*, *ATP6*, *COII*, *COIII*, *ND1*, *ND2*, *ND3*, *ND4*, *ND4L*, *ND5*, and *ND6*) is ATG, while it is ATA and ATT for *Cytb* and *COI*, respectively. Thirteen protein-coding genes had different stop codons. The stop codon for 11 genes (*ND1*, *ND4L*, *ND5*, *ND6*, *ND2*, *ND3*, *ATP8*, *Cytb*, *COI*, *COIII*, and *ATP6*) is TAR (TAA and TAG), whereas for *ND4* it is AGA. The stop codon of *COII* is a single base T, which may be finished by post-transcriptional polyadenylation which adds poly A tails to the 3′ end of mRNA (Ojala et al. 1981). The longest and shortest protein-coding genes in the mitogenome are *ND5* (1842 bp) and *ATP8* (168 bp), respectively. The 12S rRNA and 16S rRNAs are separated by the *tRNAVal* gene and are situated between the *tRNAPhe* and *tRNALeu* (UUR) genes.

We performed the phylogenetic relationship analyses to choose one species in the order Saccopharyngiformes as an outgroup. The maximum-likelihood (ML) phylogenetic tree for encoding genes (Figure 1) was constructed using the software PhyML 3.0 and the MtGTR + I+G nucleotide substitution mode. The ML phylogenetic tree of 18 Anguilliformes species indicates that *C. erebennus* is classified in the family Congridae, and the Congridae is closely related to the Nettastomatidae. Currently, Nettastomatidae is a polyphyletic group.

**Figure 1.**
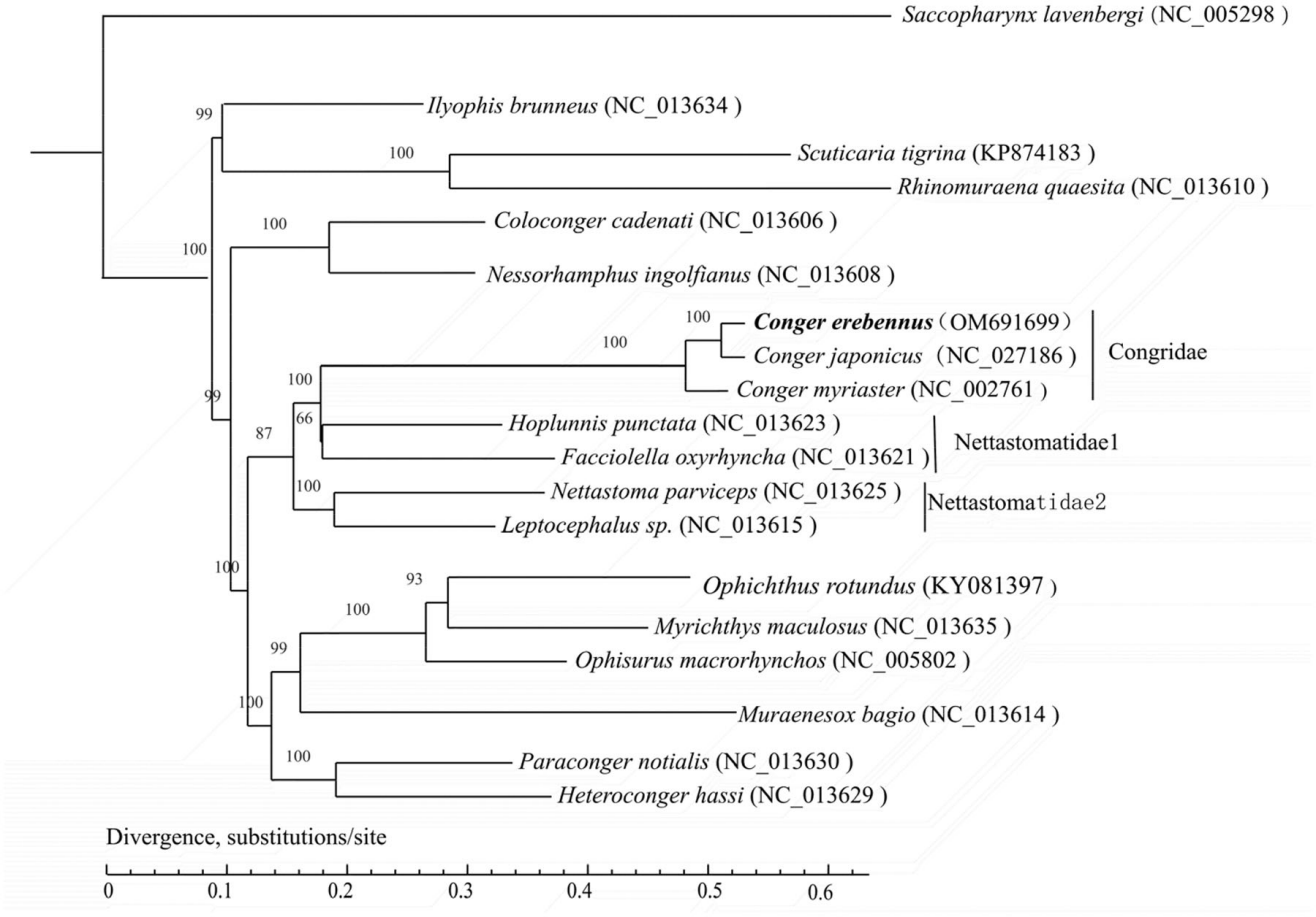
ML phylogenetic tree of *Conger erebennus* and representative Anguilliformes. The representative gene sequences are downloaded from GenBank and the bootstrap values are marked at the nodes. The encoding gene sequence of *Conger erebennus* (OM691699) is highlighted in bold font.

## Data Availability

The genome sequence data that support the findings of this study are openly available in GenBank of NCBI at https://www.ncbi.nlm.nih.gov under the accession number OM691699 or are available from the corresponding author. The associated BioProject, SRA, and Bio-Sample numbers are PRJNA805411, SRR18052220, and SAMN25860411, respectively.

## References

[CIT0001] Boore JL. 1999. Animal mitochondrial genomes. Nucleic Acids Res. 27(8):1767–1780. 10101183 10.1093/nar/27.8.1767 PMC148383

[CIT0002] Miya M, Takeshima H, Endo H, Ishiguro NB, Inoue JG, Mukai T, Satoh TP, Yamaguchi M, Kawaguchi A, Mabuchi K, et al. 2003. Major patterns of higher teleostean phylogenies: a new perspective based on 100 complete mitochondrial DNA sequences. Mol Phylogenet Evol. 26(1):121–138. 12470944 10.1016/s1055-7903(02)00332-9

[CIT0003] Odani K, Nobetsu T, Mitsuhashi M, Yabe M. 2005. Records of a conger eel, *Conger erebennus* (Actinopterygii, Anguilliformes, Congridae), from Hokkaido, Japan. Bull Biogeogr Soc Jpn. 60:21–24.

[CIT0004] Ojala D, Montoya J, Attardi G. 1981. tRNA punctuation model of RNA processing in human mitochondria. Nature. 290(5806):470–474. 7219536 10.1038/290470a0

[CIT0005] Sambrook J, Russell DW. 2001. Molecular cloning: a laboratory manual. 3rd ed. New York: Cold Spring Harbor Laboratory.

[CIT0006] Zhang K, Zhu K, Liu Y, Zhang H, Gong L, Jiang L, Liu L, Lü Z, Liu B. 2021. Novel gene rearrangement in the mitochondrial genome of *Muraenesox cinereus* and the phylogenetic relationship of Anguilliformes. Sci Rep. 11(1):2411. 33510193 10.1038/s41598-021-81622-9 PMC7844273

